# High-Performance Vacuum-Free Processed Organic Solar Cells with Gallium-Based Liquid Metal Top Electrodes

**DOI:** 10.3390/ma18122675

**Published:** 2025-06-06

**Authors:** Rui Hu, Di Xie, Yi Jin, Xiaojie Ren, Xiang Huang, Yitong Ji, Xiaotong Liu, Xueyuan Yang, Wenchao Huang

**Affiliations:** School of Materials Science and Engineering, Wuhan University of Technology, Wuhan 430070, China; 330952@whut.edu.cn (R.H.); 281533@whut.edu.cn (D.X.); 330978@whut.edu.cn (Y.J.); 331061@whut.edu.cn (X.R.); 331303@whut.edu.cn (X.H.); ytji@whut.edu.cn (Y.J.); xiaotong.liu@whut.edu.cn (X.L.); wenchao.huang@whut.edu.cn (W.H.)

**Keywords:** gallium-based liquid metals, organic solar cells, vacuum-free, all-solution-processed, ultrathin flexible

## Abstract

Conventional fabrication of high-efficiency organic solar cells (OSCs) predominantly relies on vacuum-evaporated metal top electrodes such as Ag and Al, which hinder large-scale industrial production. Gallium-based liquid metals (GaLMs), particularly the eutectic gallium–indium alloy (EGaIn), represent promising candidates to conventional vacuum-evaporated metal top electrodes due to their excellent printability and high electrical conductivity. In this study, we fabricated vacuum-free OSCs based on GaLM electrodes (Ga, EGaIn, and Galinstan) and analyzed the device performances. Rigid devices with EGaIn electrodes achieved a champion power conversion efficiency (PCE) of 15.6%. Remarkably, all-solution-processed ultrathin flexible devices employing silver nanowire (AgNW) bottom electrodes in combination with EGaIn top electrodes achieved a PCE of 13.8% while maintaining 83.4% of their initial performance after 100 compression–tension cycles (at 30% strain). This work highlights the potential of GaLMs as cost-effective, scalable, and high-performance top electrodes for next-generation flexible photovoltaic devices, paving the way for their industrial adoption.

## 1. Introduction

Organic solar cells (OSCs) have garnered significant attention in renewable energy research due to their lightweight, flexibility, and compatibility with low-cost roll-to-roll manufacturing [1,2,3,4,5]. Recent advancements in non-fullerene acceptors have boosted the PCE of single-junction OSCs with vacuum-deposited electrodes beyond 20% [6,7,8,9], demonstrating both the future prospects of OSCs and the potential for commercial applications. The fabrication of high-efficiency OSCs employing vapor-deposited metal conductors as top electrodes is energy-intensive and incompatible with large-area production, limiting their applicability in wearable electronics and internet-of-things (IoT) devices [10,11,12]. Consequently, developing low-cost printable electrodes for vacuum-free, all-solution-processed OSCs has become imperative.

To address this challenge, solution-processable electrode materials have been extensively explored [13,14,15,16,17,18]. For the bottom electrode, PEDOT:PSS and AgNWs, with their high transmittance and excellent solution processability, are ideal candidates to replace magnetron-sputtered indium tin oxide (ITO) electrodes [19,20,21,22,23]. In contrast, top electrodes increasingly utilize low-melting-point alloys, such as Field’s metal and gallium-based liquid metals (GaLMs), due to their high electrical conductivity and optical reflectivity [24,25]. Among these electrodes, GaLMs exhibit unique advantages, including room-temperature liquidity, low toxicity, non-volatility, low work function, and biocompatibility, making them ideal for soft electronics and biomedical applications [26,27,28,29]. These properties also make GaLMs a promising candidate for novel electrode materials that can circumvent the need for high-vacuum deposition processes.

Commonly used GaLMs include Ga, Ga_75.5_In_24.5_ (eutectic gallium−indium or EGaIn), and Ga_68.5_In_21.5_Sn_10_ (Galinstan) [30]. These liquid metal materials have been successfully applied in photovoltaic applications. For instance, Galinstan was used as an interface modifier material in combination with a carbon electrode to facilitate hole extraction [31]. Furthermore, LM/carbon composite electrodes were prepared by uniformly dispersing liquid gallium droplets into carbon electrodes to enhance the electrical conductivity and interfacial contact for more efficient charge transfer in perovskite solar cells [32]. Notably, EGaIn-coated OSCs achieve a PCE of 14.1% without electron transport layers [33]. Additionally, the stretchable OSCs prepared by spray-deposited EGaIn electrodes exhibit excellent mechanical robustness [34,35,36,37,38].

GaLMs can replace evaporated metal electrodes, offering greater convenience in the fabrication process, albeit with relatively lower efficiency. A comprehensive investigation into the influence of GaLMs with varying compositions (from single-component to multi-component alloys) on device performance and underlying physics is of significant importance for further improving the efficiency of OSCs based on liquid metal top electrodes. In this work, we systematically compare Ga, EGaIn, and Galinstan as top electrodes for vacuum-free OSCs. Among the three different top electrodes, EGaIn-based devices exhibited superior charge transport kinetics, suppressed recombination, and a record PCE of 15.6%. Furthermore, ultrathin flexible OSCs fabricated via all-solution processing with EGaIn top electrodes and AgNW bottom electrodes achieved a PCE of 13.8% and retained 83.4% of initial efficiency after 100 compression–tension cycles at 30% strain, demonstrating excellent mechanical resilience for wearable applications.

## 2. Experimental

### 2.1. Materials

PM6, D18, D18-Cl, L8-BO, Y6, BTP-eC9, and PDINN were purchased from Solarmer Materials Inc. (Beijing, China). Poly(3,4-ethylenedioxythiophene):poly(styrene sulfonate) (PEDOT:PSS, Clevios™ PVP 4083) aqueous solution was obtained from Heraeus (Hanau, Germany). Silver nanowires (AgNWs) dispersed in isopropanol (IPA) (10 mg mL^−1^, with an average length of 10–30 μm and diameter of 20–30 nm) were supplied by Zhejiang Kechuang Advanced Materials Co., Ltd. (Zhejiang, China). Indium tin oxide (ITO)-coated glass substrates were procured from Suzhou Sunyang Solar Technology Co. (Suzhou, China). Gallium (Ga, 99.99%), indium (In, 99.99%), tin (Sn, 99.98%) and other chemicals were acquired from Aladdin Reagent Co. (Shanghai, China) and used as received. Eutectic gallium–indium (EGaIn) was prepared by mixing 75.5 wt% Ga and 24.5 wt% In at 100 °C for 2 h under a nitrogen atmosphere. Galinstan (68.5 wt% Ga, 21.5 wt% In, 10 wt% Sn) was prepared using an analogous protocol.

### 2.2. Device Fabrication

The rigid OSCs were fabricated in the structure of ITO/PEDOT:PSS/active layer/PDINN/GaLMs. First, ITO glass substrates were ultrasonically cleaned sequentially in deionized water, isopropanol, and ethanol (10 min each), then dried with a nitrogen gun. Next, substrates were plasma treated for 15 min, and then the PEDOT:PSS solution was spin-coated at 5000 rpm for 40 s, followed by annealing on a hot plate at 150 °C for 10 min. The active layer solution of PM6:L8-BO (1:1.2 *w*/*w* with a total concentration of 16.5 mg mL^−1^) in the solvent of chloroform with 1, 4-diiodobenzene (DIB) additive was spin-coated at 3500 rpm for 30 s, followed by annealing at 100 °C for 5 min. Then, a PDINN solution with a concentration of 0.8 mg mL^−1^ was spin-coated at 3000 rpm for 30 s. Finally, the GaLM was spray-coated onto the PDINN layer through a shadow mask with a nitrogen gas pressure of 0.4 MPa and a spraying height of 30 cm. The effective active area of the OSCs was 0.04 cm^2^.

To fabricate the all-solution-processed ultrathin flexible OSC, a clean glass substrate was treated in plasma for 15 min. Then, the fluorinated polymer layer (Novec 1700:7100) was spin-coated on the glass substrate at 4000 rpm. A parylene film was prepared through chemical vapor deposition, and then AgNW solution (concentration of 2 mg mL^−1^) was sprayed onto the substrate to form patterned electrodes, with a spraying height of 20 cm and a spraying N_2_ pressure of 0.06 MPa. Subsequent layers were deposited using the same procedure as for rigid devices.

### 2.3. Characterizations

Absorption, transmission and reflectivity spectra were recorded using a Shimadzu UV-1900i spectrophotometer (Shimadzu Corporation, Kyoto, Japan). Film thickness was measured by a Dektak XT probe profiler (produced by Bruker, Billerica, MA, USA). The sheet resistances of samples were measured using the 4 Point Probes Measurement System (HPS2524). Photovoltaic parameters and mechanical stability of OSCs with EGaIn cathodes were tested in a nitrogen glove box. Current density–voltage (J–V) curves were acquired under AM 1.5G illumination (100 mW cm^−2^), using a Keithley 2450 SourceMeter (Keithley Instruments, OH, USA). The device area of 0.04 cm^2^ was defined by using a shadow mask. All photovoltaic parameters of devices were averaged over 16 independent devices. The light intensity was calibrated using a standard silicon reference cell certified by the National Renewable Energy Laboratory (NREL, Golden, CO, USA). The EQE was obtained using a solar cell spectral response measurement system (Enli Technology Co., Ltd., Kaohsiung City, Taiwan, QE-R). The light intensity of the system was calibrated by the reference Si probe (RC-S103011-E) obtained from Enli Technology Co., Ltd. The mechanical stability of ultrathin OSCs was averaged over 6 devices. Mechanical stability of the OSCs was tested using an automated tensile-bending tester (PR-BDM4-100V, Shenzhen Purui Materials Technology Co., Ltd., Shenzhen, China). Transient photocurrent (TPC) and photovoltage (TPV) were measured with a LST-TPC system (Shanghai Jinzhu Technology Co., Ltd., Shanghai, China). Scanning electron microscopy (SEM) images were acquired with a JEOL JSM-7500F microscope (JEOL Ltd., Tokyo, Japan). Atomic force microscopy (AFM) was performed on a Cypher ES system (Asylum Research, High Wycombe, UK).

## 3. Results and Discussion

GaLMs without the surface oxide layer exhibit high surface tension values of 727 mN m^−1^ for Ga (at 30 °C) [39], 624 mN m^−1^ for EGaIn (at 22 °C) [40], and 533 mN m^−1^ for Galinstan (at 25 °C) [41], respectively. These properties inherently limit their wettability on non-metallic substrates. As shown in Figure S1, GaLMs exhibit poor wettability on the electron transport layer (ETL) film, with metallic Ga demonstrating the worst wetting behavior among them. However, spontaneous oxidation in the ambient atmosphere forms a thin oxide layer, significantly enhancing GaLM–substrate adhesion and interfacial compatibility. Therefore, in this work, GaLMs are deposited by spraying in air, which facilitates wetting with the ETL. 

**Figure 1:**
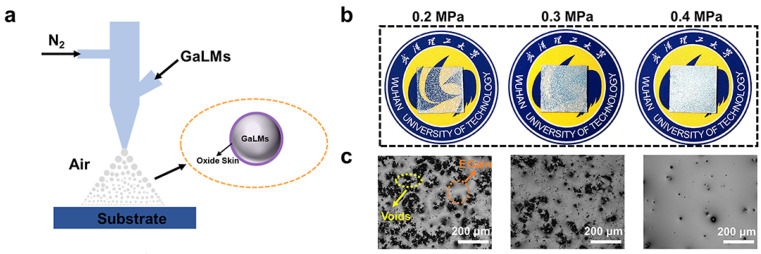
Thin GaLM film preparation. (**a**) Experimental setup for spraying GaLMs. (**b**) The physical pictures and (**c**) optical microscope images of EGaIn sprayed under gas pressures of 0.2, 0.3, and 0.4 MPa, respectively.

To determine the optimal spray pressure, customized spray-coating equipment was developed with tunable pressure, as illustrated in Figure 1a. Film morphology analysis revealed that a pressure of 0.4 MPa was optimal for forming continuous GaLM films (Figure 1b,c). When the pressure is too low (0.2–0.3 MPa), GaLMs are difficult to eject, resulting in porous structures with micron-scale voids. When the nitrogen pressure reaches 0.4 MPa, the large holes predominantly vanish, ensuring uniform coverage. The incomplete elimination of voids may result from the entrapment of high-velocity nitrogen gas during film deposition [42]. To mitigate nozzle clogging, a common issue due to Ga’s melting point (29.8 °C) near room temperature, both the spray gun and GaLM precursor are preheated to 50 °C prior to deposition.

**Figure 2:**
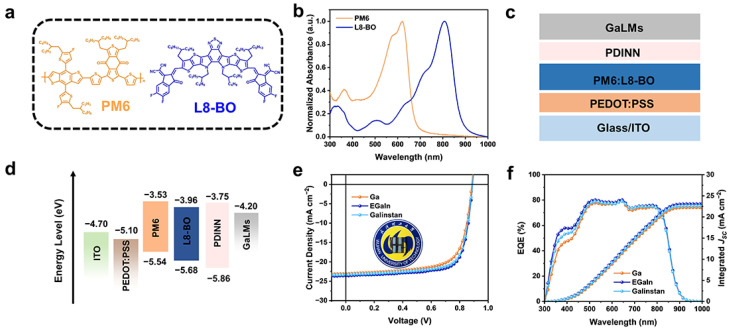
Device performance of OSCs with GaLM top electrodes. (**a**) Chemical structures of PM6 and L8-BO. (**b**) Normalized UV–vis spectra of the pristine PM6 and L8-BO films. (**c**) The device structure of OSCs. (**d**) Energy level diagram. (**e**) *J*–*V* curves. (**f**) EQE spectra and integrated *J_SC_*.

To prepare high-performance OSCs, the photoactive layer comprised the donor PM6 and acceptor L8-BO, and their molecular structures are depicted in Figure 2a. The UV–vis absorption spectra of pristine PM6 and L8-BO films are presented in Figure 2b. The absorption spectrum of the PM6 donor material complements well with that of L8-BO in the range of 500–900 nm, enabling broad-spectrum light harvesting. Devices with the architecture ITO/PEDOT:PSS/PM6:L8-BO/PDINN/GaLM (Figure 2c) were fabricated. The energy level alignment is illustrated in Figure 2d, where the EGaIn cathodes with low work function facilitate efficient electron collection from the lowest unoccupied molecular orbital of the acceptor. To evaluate the effects of GaLM top electrodes on the photovoltaic performance of OSCs, the devices were fabricated by depositing three types of GaLMs. Here, the representative EGaIn electrode was used to optimize the spraying pressure and height, which were set at 0.4 MPa and 30 cm, respectively (Figures S2 and S3 and Tables S1 and S2). Figure 2e shows the *J–V* curves under AM 1.5G solar irradiation, and the corresponding performance parameters are summarized in Table 1. The device with the EGaIn top electrode achieves a PCE of 15.6% with an open-circuit voltage (*V_OC_*) of 0.890 V, a short-circuit current density (*J_SC_*) of 23.6 mA cm^−2^, and a fill factor (FF) of 74.5%, which outperforms the other two GaLM counterparts. The PCEs are 14.4% and 15.3% for Ga-OSC and Galinstan-OSC, respectively. The external quantum efficiency (EQE) spectrum is shown in Figure 2f, which shows the consistency of the short-circuit current pattern with less than 5% deviation from that of a solar simulator test. Meanwhile, OSCs prepared with EGaIn exhibit the highest EQE value, which contributes to the enhancement of the *J_SC_* and the PCE. The enhanced EQE performance observed in EGaIn-based devices originates from the highest optical reflectivity of the EGaIn electrodes (Figure S4). In addition, the stability of devices incorporating GaLM electrodes was evaluated under nitrogen atmosphere at 100 °C (Figure S5). Remarkably, devices with EGaIn electrodes demonstrated superior stability, retaining 80.1% of their initial PCE after 600 min.

**Figure 3:**
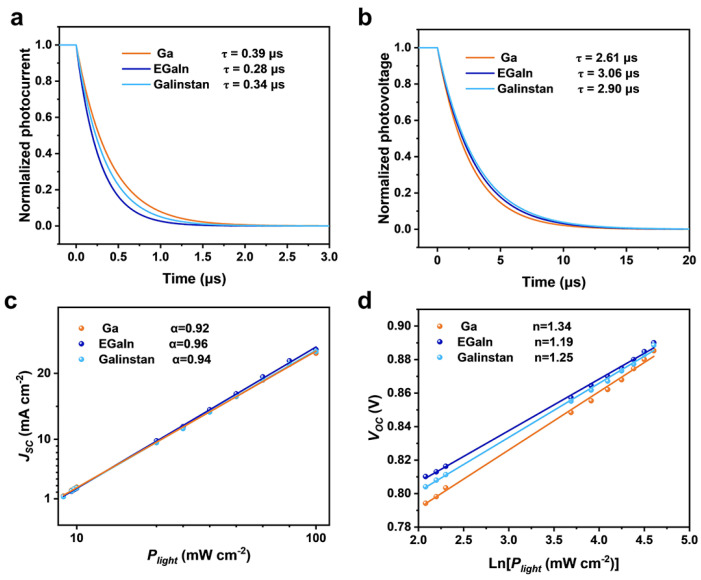
Device physics of the OSCs with different top electrodes. (**a**) Normalized transient photocurrent (TPC) decay for the OSCs. (**b**) Normalized transient photovoltage (TPV) decay for the OSCs. (**c**) The dependence of *J_SC_* on light intensity. (**d**) The dependence of *V_OC_* on light intensity.

To investigate the collection and recombination of charge carriers in the LM-electrode OSCs, transient photocurrent (TPC) and transient photovoltage (TPV) tests were conducted to quantify charge extraction and recombination (Figure 3a,b). By fitting the TPC curves, it can be seen that the charge extraction time of the OSCs based on EGaIn electrodes is 0.28 μs, faster than the extraction speeds of the Ga and Galinstan electrodes (0.39 μs and 0.34 μs, respectively). Moreover, the carrier lifetimes obtained from the TPV decay dynamics are 2.61 μs, 3.06 μs, and 2.90 μs for the Ga-OSC, EGaIn-OSC, and Galinstan-OSC, respectively. The longer carrier lifetime suggests reduced charge recombination in the EGaIn device.

Bimolecular recombination was investigated by fitting the photocurrent *J_SC_* to the light intensity (*P_light_*) by the scaling of *J_SC_* ∝ (*P_light_*)^α^. As shown in Figure 3c, the EGaIn devices exhibit higher α values (0.96) than those of the Ga devices and Galinstan devices (0.92 and 0.94, respectively), indicating lower bimolecular recombination for EGaIn devices. Furthermore, the relationship between *Voc* and *P_light_* can be determined by the linear relationship *Voc* ∝ (nkT q^−1^) ln(*P_light_*) to reveal trap-assisted recombination, where k is the Boltzmann constant, T is the absolute temperature, and q is the elementary charge. Devices based on EGaIn top electrodes exhibit a slope of 1.19 kT q^−1^, which is lower than other GaLM devices (1.34 kT q^−1^ for the Ga-cathode and 1.25 kT q^−1^ for the Galinstan-cathode). The results demonstrate less trap-assisted recombination in the EGaIn devices. Furthermore, the dark *J*–*V* curves of GaLM-OSCs are shown in Figure S6, and the EGaIn devices exhibit a lower leakage current.

Moreover, the interfacial contact quality between the liquid metal and PDINN plays a critical role in determining charge transport efficiency and recombination dynamics. Therefore, to evaluate the quality of the contact interfaces formed between different liquid metals and the ETL, we conducted a comparative analysis of the series resistance (*R_s_*) and shunt resistance (*R_sh_*) in the corresponding devices. As shown in Figure S7, the OSCs with EGaIn electrodes exhibited the lowest *R_s_*, which is beneficial for charge transport, while the high *R_sh_* effectively suppresses shunt losses, thereby reducing current leakage and recombination processes [43,44,45].

**Figure 4:**
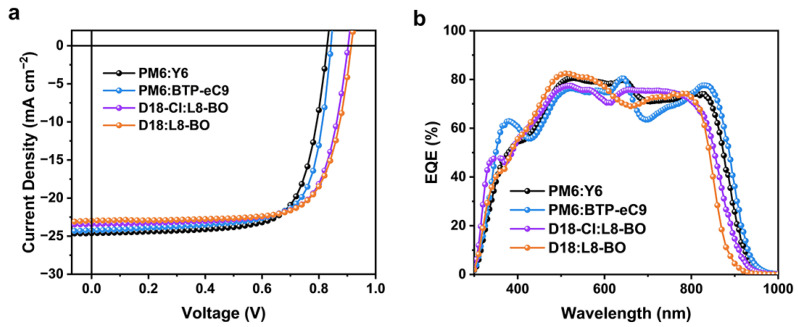
Performance of OSCs with EGaIn top electrodes based on different active layer systems. (**a**) *J*–*V* curves. (**b**) EQE spectra.

To verify the universality of EGaIn top electrodes, OSCs with different active layers were fabricated and characterized. The molecular structures of the active layer materials and their corresponding absorption spectra are depicted in Figure S8, associated with the corresponding energy level diagram (Figure S9). Notably, all EGaIn devices demonstrate remarkable PCEs. The *J*–*V* curves of these devices are illustrated in Figure 4a, and the EQE spectra of the devices are shown in Figure 4b, with detailed photovoltaic parameters summarized in Table 2. This consistency underscores EGaIn’s versatility as a high-performance top electrode for OSCs.

**Figure 5:**
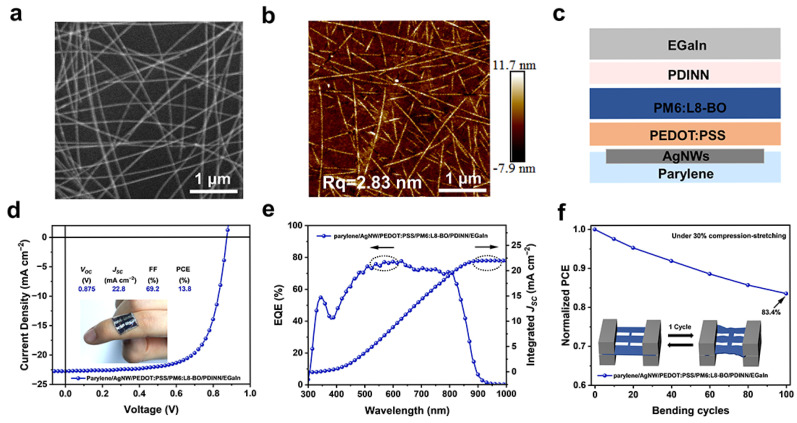
All-solution-processed OSCs based on the AgNW anode and EGaIn cathode. (**a**) Scanning electron microscopy (SEM) images and (**b**) atomic force microscopy (AFM) of parylene/AgNW substrate. (**c**) Device architecture of the all-solution-processed ultrathin flexible OSC. (**d**) *J−V* curves of the all-solution-processed ultrathin flexible OSC under AM 1.5G illumination at 100 mW cm^−2^. (**e**) EQE spectra of the all-solution-processed ultrathin flexible OSC. (**f**) Mechanical stability of all-solution-processed ultrathin flexible OSC under cyclic compression/stretching test.

To fabricate fully vacuum-free OSCs, all-solution-processed flexible devices were developed by using parylene/AgNW as the transparent bottom electrode. As shown in Figure S10, the parylene/AgNW electrode exhibits excellent optical transmittance. The scanning electron microscope (SEM) and atomic force microscope (AFM) images of the parylene/AgNW electrode are shown in Figure 5a,b. SEM characterization clearly demonstrates that AgNWs form an interconnected conductive network through their cross-linked architecture. The AFM analysis reveals a surface roughness (Rq) of 2.83 nm, indicating a smooth morphology suitable for subsequent device fabrication. Notably, the parylene/AgNW electrode demonstrates an ultrathin profile with a thickness of approximately 1 μm. To fabricate all-solution-processed ultrathin flexible OSCs, the bottom transparent electrode is parylene/AgNW, combined with EGaIn as the top electrode. The device was fabricated based on the structure of parylene/AgNW/PEDOT:PSS/PM6:L8-BO/PDINN/EGaIn, illustrated in Figure 5c. The *J*–*V* curves of the all-solution-processed ultrathin flexible OSC are shown in Figure 5d, exhibiting excellent device performance with a *V_OC_* of 0.875 V, *J_SC_* of 22.8 mA cm^−2^, FF of 69.2%, and PCE of 13.8%. The EQE spectrum of the device is depicted in Figure 5e. The ultrathin flexible OSCs, after being peeled off from the glass substrate, were transferred onto the pre-stretched elastomeric material (3M VHB). The peeling-off process does not significantly alter the device performance, confirming the reliability and practical applicability of the ultrathin OSC (Figure S11 and Table S3). After releasing the prestretch, the ultrathin OSC exhibits a buckling structure. The mechanical stability of the ultrathin OSCs was measured by a cyclic compression/stretching test with an actual compression rate of 30%. The EGaIn electrode exhibited stable electrical conductivity under the cyclic compression/stretching test, whereas the AgNW electrode demonstrated notable degradation in conductive performance (Figure S12). As shown in Figure 5f, the ultrathin flexible OSC maintains 83.4% of its initial efficiency after 100 compression/stretching cycles, further demonstrating the excellent mechanical properties of the device and showing great potential for applications in wearable devices.

## 4. Conclusions

In this study, gallium-based liquid metals (GaLMs) were systematically investigated as top electrodes for vacuum-free organic solar cells (OSCs). Among the evaluated GaLMs (Ga, EGaIn, and Galinstan), the OSCs based on EGaIn top electrodes demonstrate superior performance, confirmed by improved charge extraction and suppressed carrier recombination. The rigid PM6:L8-BO OSC based on EGaIn cathodes achieves an efficiency of 15.6%. Furthermore, the ultrathin flexible OSC fabricated via all-solution processing with AgNW bottom transparent electrodes and EGaIn top electrodes exhibits a PCE of 13.8%, while retaining 83.4% of initial efficiency after 100 compression–stretching cycles, demonstrating exceptional mechanical resilience for wearable applications. The long-term operational stability of ultrathin flexible organic solar cells (OSCs) constitutes a critical parameter that fundamentally determines their commercial viability in emerging photovoltaic markets. Furthermore, minimizing efficiency losses during the scaling process from laboratory-scale devices to industrially relevant large-area flexible mini-modules remains an essential prerequisite for practical implementation in wearable electronic systems.

## Figures and Tables

**Table 1:** Photovoltaic parameters of the devices based on different GaLM electrodes.

Top Electrodes	*V_OC_* (V)	*J_SC_* (mA cm^−2^)	*Cal.J_SC_* (mA cm^−2^)	FF (%)	PCE_max_ (%)	PCE_avg_ ± SD (%)
Ga	0.885	23.1	22.3	70.2	14.4	14.0 ± 0.4
EGaIn	0.890	23.6	23.0	74.5	15.6	15.4 ± 0.2
Galinstan	0.889	23.3	22.6	73.6	15.3	15.0 ± 0.3

Average values with standard deviations in parentheses are calculated from 16 individual devices.

**Table 2:** Photovoltaic parameters of the devices based on different active layer systems.

Active Layer	*V_OC_* (V)	*J_SC_* (mA cm^−2^)	FF (%)	PCE_max_ (%)	PCE_avg_ ± SD (%)
PM6:Y6	0.829	24.6	72.3	14.7	14.3 ± 0.4
PM6:BTP-eC9	0.842	24.5	73.1	15.1	14.6 ± 0.5
D18:L8-BO	0.914	23.0	73.6	15.5	15.1 ± 0.4
D18-Cl:L8-BO	0.903	23.4	72.9	15.4	15.0 ± 0.4

Average values with standard deviations in parentheses are calculated from 16 individual devices.

## Data Availability

The original contributions presented in this study are included in the article/Supplementary Material. Further inquiries can be directed to the corresponding author.

## Supplementary Materials

The following supporting information can be downloaded at: https://www.mdpi.com/article/10.3390/ma18122675/s1, Figure S1: The contact angle images of GaLMs on the PDINN film; Figure S2: Device performance of the devices fabricated by spray-coating EGaIn under different nitrogen gas pressures. (a) *J*–*V* curves and (b) PCE as a function of nitrogen gas pressures; Figure S3: Device performance of the devices fabricated by spray-coating EGaIn at varying heights. (a) *J*–*V* curves and (b) PCE as a function of spraying heights; Figure S4: The reflectance spectra of GaLM electrodes; Figure S5: Storage stability of the OSCs based on different GaLM electrodes; Figure S6: Dark *J*–*V* curves for the OSCs based on different GaLM electrodes; Figure S7: *R_s_* and *R_sh_* of OSCs with GaLM top electrodes; Figure S8: (a) Chemical structures of D18, D18-Cl, Y6 and BTP-eC9. (b) Normalized UV−vis absorption spectra of the PM6:Y6, PM6: BTP-eC9, D18-Cl:L8-BO, and D18:L8-BO films; Figure S9: Energy level diagram; Figure S10: Optical transmittance spectra of the parylene/AgNW electrode; Figure S11: *J*–*V* curves of the ultrathin flexible device before and after the peeling-off process; Figure S12: The conductive performance of the Ga, EGaIn, Galinstan, and AgNW electrodes. (a) Electrical conductivity and (b) the normalized *R_sh_* under under cyclic compression/stretching test; Table S1: Photovoltaic parameters of the devices fabricated by spray-coating EGaIn under different nitrogen gas pressures measured under 100 mW cm^−2^; Table S2: Photovoltaic parameters of the devices fabricated by spray-coating EGaIn at varying heights; Table S3: Photovoltaic parameters of the ultrathin flexible device before and after the peeling-off process.
