# Supplementary material for: High-Performance Vacuum-Free Processed Organic Solar Cells with Gallium-Based Liquid Metal Top Electrodes
Source: Materials (Basel). 2025 Jun 6;18(12):2675. doi: 10.3390/ma18122675 (PMC12194084; doi:10.3390/ma18122675)
Supplement: Supplementary file 1 [file materials-18-02675-s001.zip › materials-3584737-supplementary.pdf]

Supplementary Materials

High-Performance Vacuum-Free Processed Organic Solar Cells with Gallium-Based Liquid Metal Top Electrodes

Rui Hu[1], Di Xie[1], Yi Jin[1], Xiaojie Ren[1], Xiang Huang[1], Yitong Ji[1], Xiaotong Liu[1], Xueyuan Yang[1]*, Wenchao Huang[1]

[1] School of Materials Science and Engineering, Wuhan University of Technology, Wuhan, 430070, China

* Correspondence: xueyuan.yang@whut.edu.cn (X.Y.)

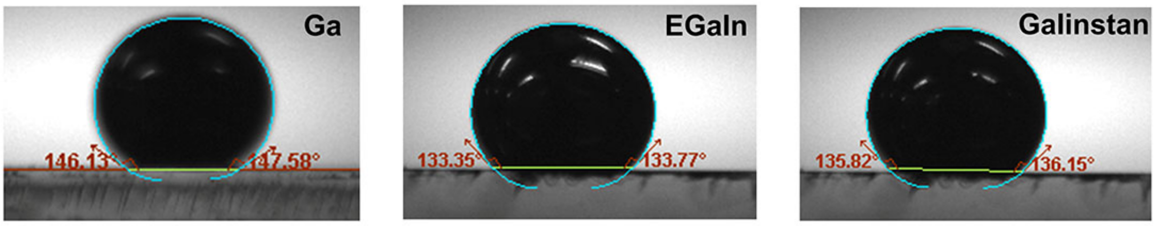

Figure S1. The contact angle images of GaLMs on the PDINN film.

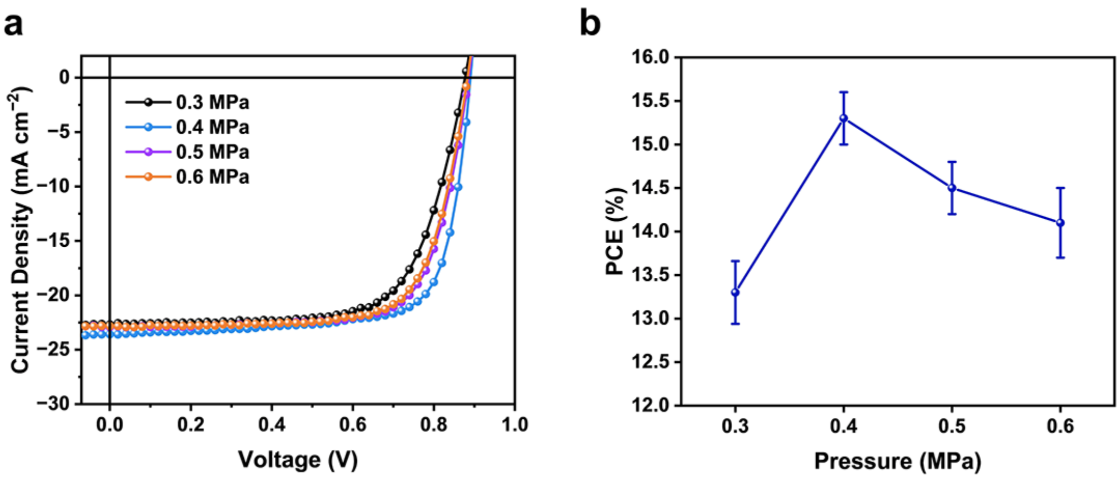

Figure S2. Device performance of the devices fabricated by spray-coating EGaIn under different nitrogen gas pressures. (a) *J–V* curves and (b) PCE as a function of the nitrogen gas pressures.

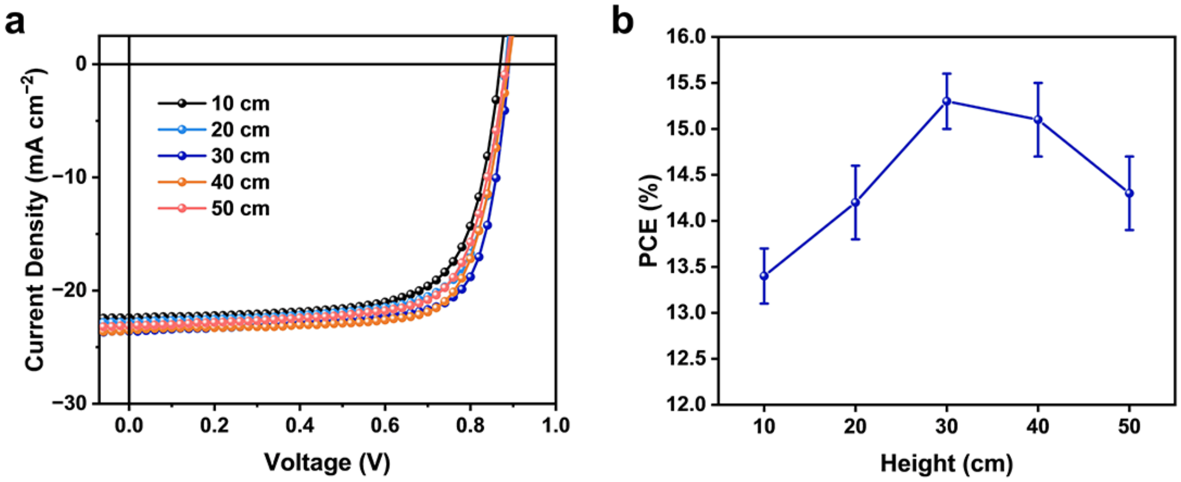

Figure S3. Device performance of the devices fabricated by spray-coating EGaIn at varying heights. (a) *J–V* curves and (b) PCE as a function of spraying heights.

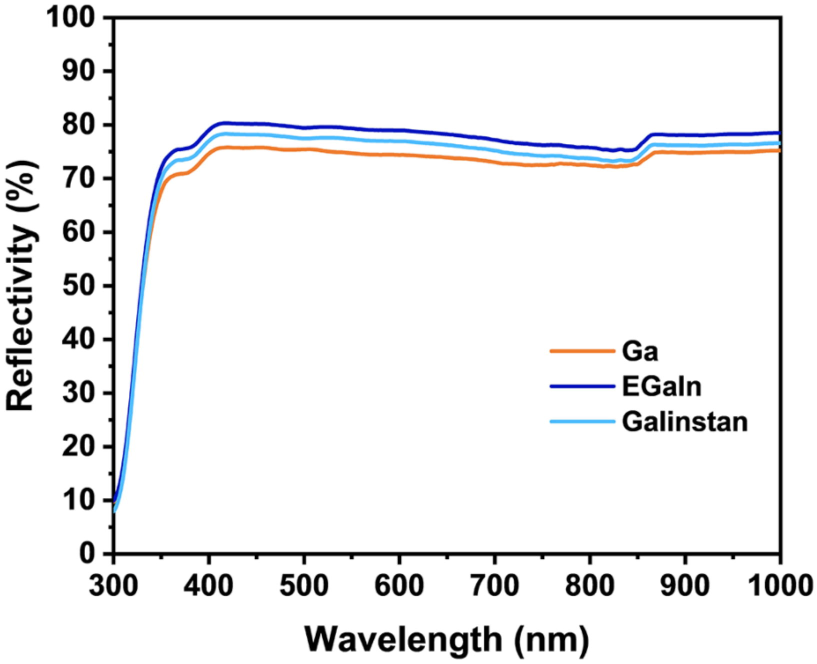

Figure S4. The reflectance spectra of GaLM electrodes.

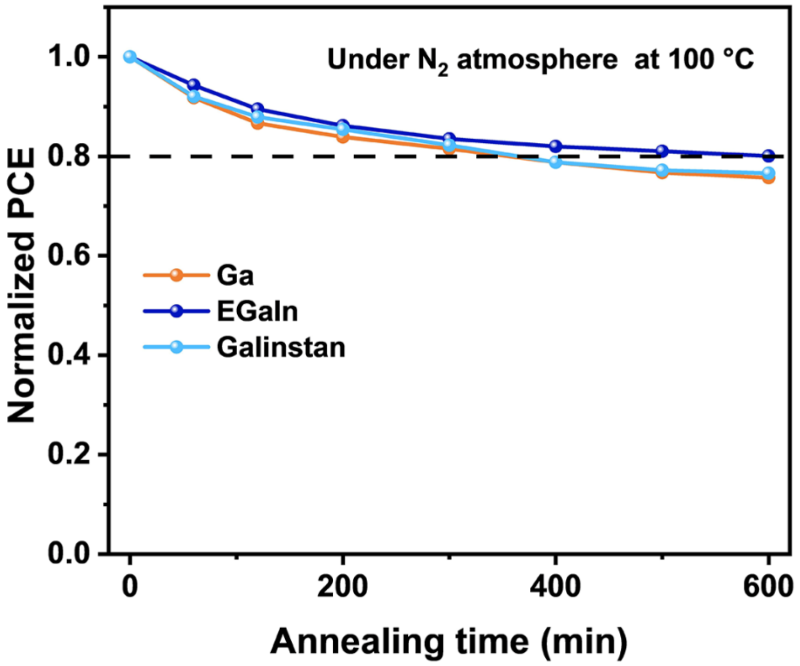

Figure S5. Thermal stability of the OSCs based on different GaLM electrodes annealed at 100 °C.

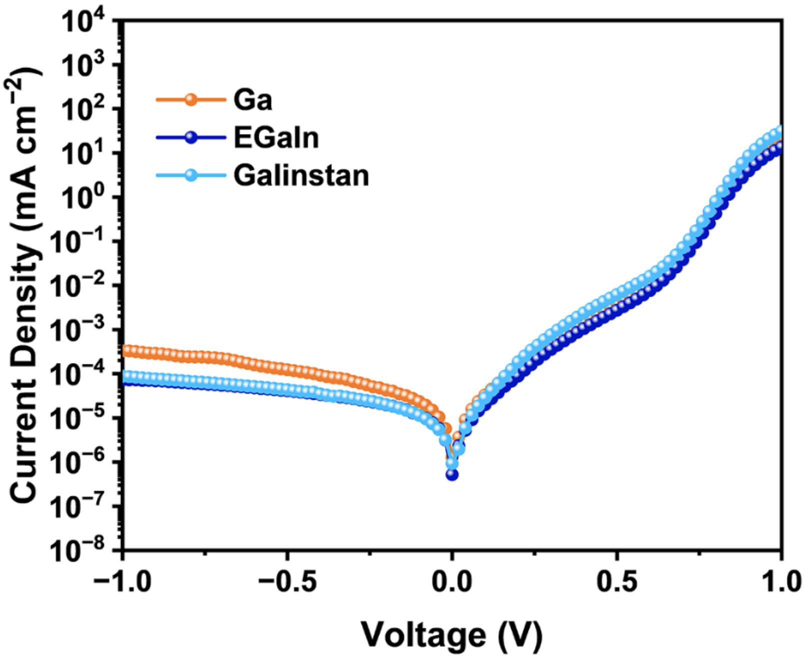

Figure S6. Dark *J*–*V* curves for the OSCs based on different GaLM electrodes.

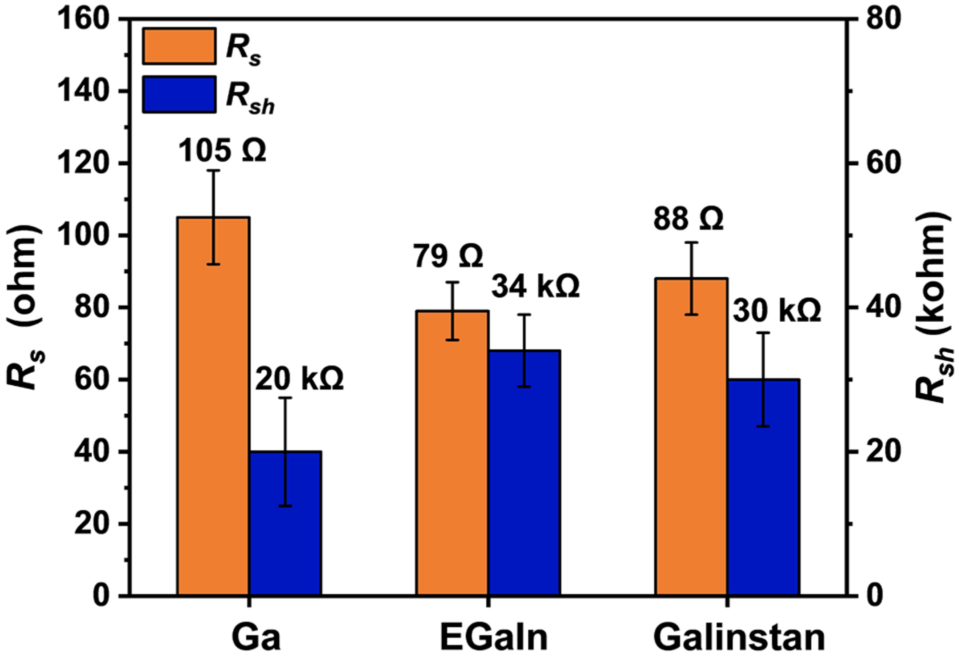

Figure S7. R_s and R_{sh} of OSCs with GaLM top electrodes.

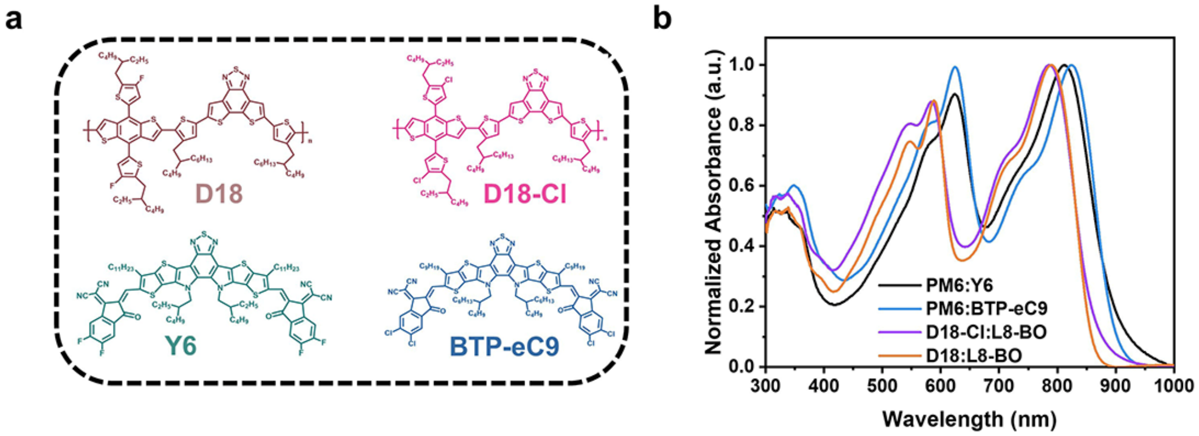

Figure S8. (a) Chemical structures of D18, D18-Cl, Y6 and BTP-eC9. (b) Normalized UV−vis absorption spectra of the PM6:Y6, PM6: BTP-eC9, D18-Cl:L8-BO, and D18:L8-BO films.

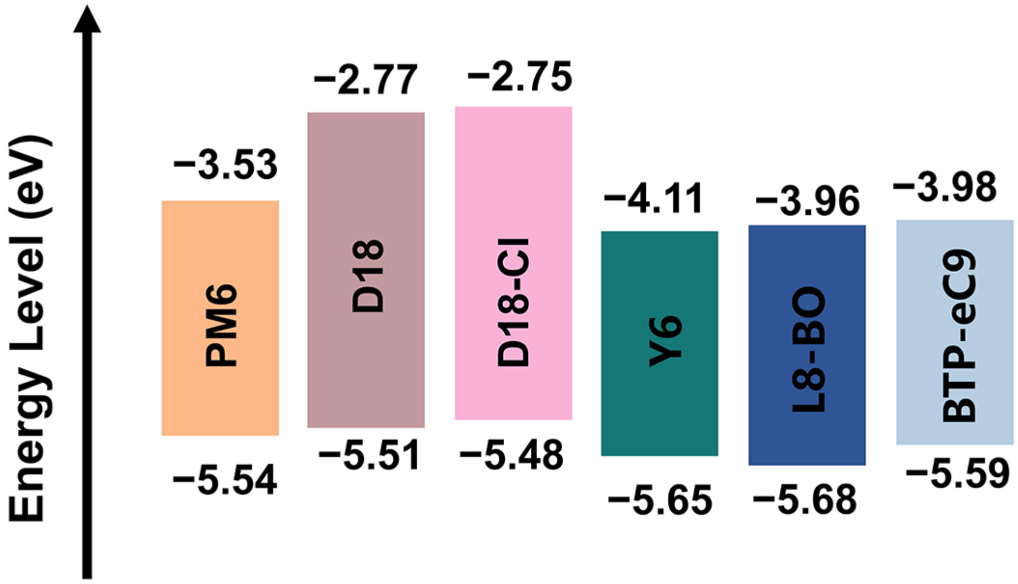

Figure S9. Energy level diagram.

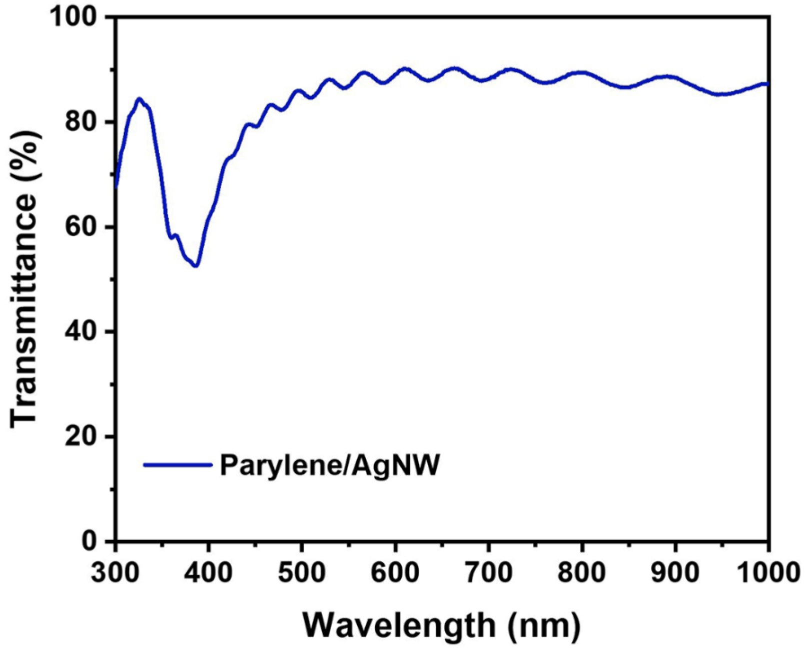

Figure S10. Optical transmittance spectra of the parylene/AgNW electrode.

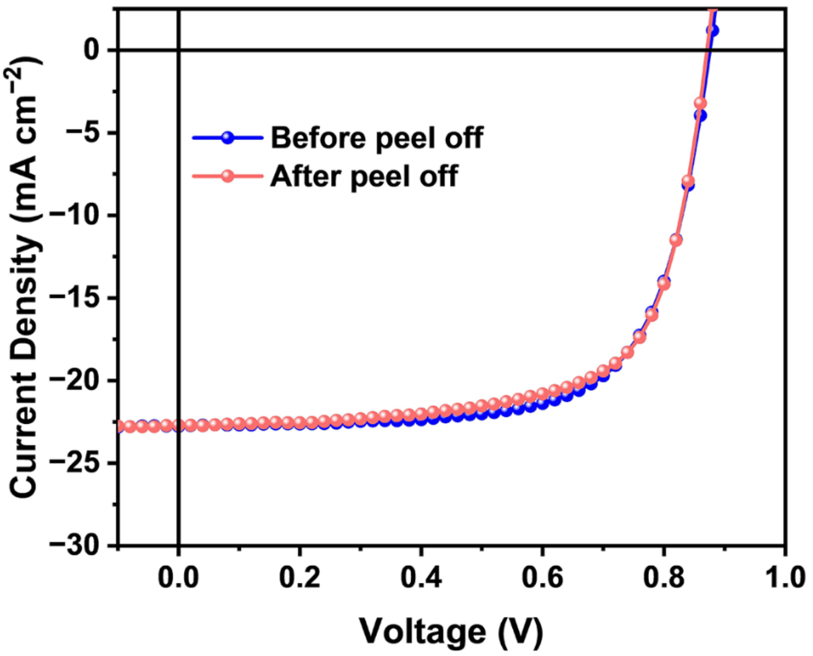

Figure S11. *J*–*V* curves of the ultrathin flexible device before and after the peeling-off process.

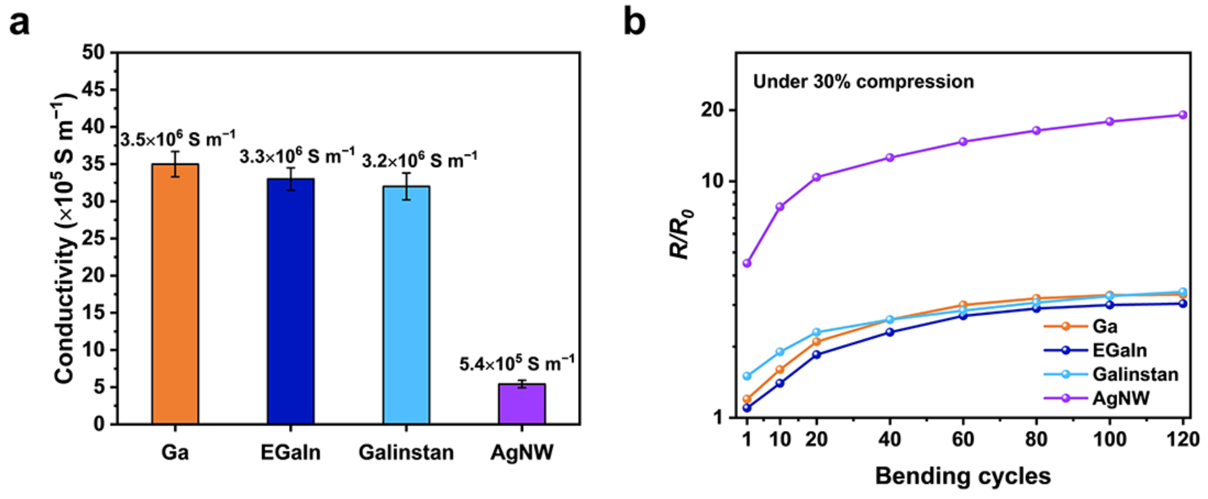

Figure S12. The conductive performance of the Ga, EGaIn, Galinstan, and AgNW electrodes. (a) Electrical conductivity and (b) the normalized R_{sh} under under cyclic compression/stretching test.

Table S1. Photovoltaic parameters of the devices fabricated by spray-coating EGaIn under different nitrogen gas pressures measured under 100 mW cm^{-2}.

Pressure (MPa)	V_{OC} (V)	J_{SC} (mA cm^{-2})	FF (%)	$PCE_{max(avg)}$ (%)
0.3	0.876 (0.871±0.005)	22.6 (22.2±0.4)	69.0 (68.4±0.6)	13.7 (13.2±0.5)
0.4	0.891 (0.888±0.003)	23.6 (23.3±0.3)	74.4 (74.0±0.4)	15.6 (15.3±0.3)
0.5	0.885 (0.881±0.004)	22.9 (22.6±0.3)	73.1 (72.6±0.5)	14.8 (14.5±0.3)
0.6	0.882 (0.878±0.004)	22.8 (22.4±0.4)	72.3 (71.8±0.5)	14.5 (14.1±0.4)

Average values with standard deviations in parentheses are calculated from 16 individual devices.

Table S2. Photovoltaic parameters of the devices fabricated by spray-coating EGaIn at varying heights.

Spraying height (cm)	V_{OC} (V)	J_{SC} (mA cm^{-2})	FF (%)	$PCE_{max(avg)}$ (%)
10	0.870 (0.865±0.005)	22.4 (22.0±0.4)	70.5 (70.0±0.5)	13.7 (13.3±0.4)
20	0.883 (0.879±0.004)	22.8 (22.4±0.4)	72.4 (72.0±0.4)	14.6 (14.2±0.4)
30	0.890 (0.887±0.003)	23.5 (23.2±0.3)	74.6 (74.2±0.4)	15.6 (15.3±0.3)
40	0.890 (0.886±0.004)	23.5 (23.1±0.4)	74.1 (73.6±0.5)	15.5 (15.1±0.4)
50	0.883 (0.878±0.005)	23.1 (22.7±0.4)	72.0 (71.5±0.5)	14.7 (14.3±0.4)

Average values with standard deviations in parentheses are calculated from 16 individual devices.

Table S3. Photovoltaic parameters of the ultrathin flexible device before and after the peeling-off process.

	V_{OC} (V)	J_{SC} (mA cm^{-2})	FF (%)	PCE (%)
Before peeling-off	0.875	22.8	69.2	13.7
After peeling-off	0.867	22.8	68.2	13.5
